# Interpretable intrusion detection for IoT: a CNN-BiLSTM permutation importance framework for deep feature selection

**DOI:** 10.3389/fdata.2026.1813265

**Published:** 2026-05-22

**Authors:** Ibrahim Al-Shibly, Llorenç Burgas, Joaquim Massana

**Affiliations:** Control Engineering and Intelligent Systems, Universitat de Girona, Campus Montilivi, EPS IV, Girona, Catalonia, Spain

**Keywords:** CIC IIoT 2025, CNN–BiLSTM, cybersecurity, deep feature selection, IIoT intrusion detection, machine learning

## Abstract

Industrial intrusion detection systems (IDS) in Industrial Internet of Things (IIoT) environments have to address the problem of handling multi-feature temporally correlated network traffic and dynamic changes in attack patterns. Traditional filter-based feature selection methods, like Mutual Information (MI), only consider individual feature performance and may not be effective in dealing with non-linear feature dependencies. This may degrade detection performance, especially in class-imbalanced problems. To mitigate such challenges, this paper proposes a deep feature selection (DFS) framework that utilizes a hybrid Convolutional Neural Network (CNN) and Bidirectional Long Short-Term Memory (BiLSTM) model. The proposed framework assesses the importance of native features using permutation importance. In the proposed framework, the CNN model detects local features in the data, whereas the BiLSTM model detects bidirectional temporal features in the data. The importance of features is computed by assessing the performance degradation of the model using time-aware perturbations on individual features. These identified features that are most relevant are then used to train lightweight traditional machine learning models like decision tree, K-nearest neighbor (KNN), logistic regression, naïve Bayes, and random forest. This makes it easy to deploy in resource-constrained IIoT environments. The approach is tested on the CIC IIoT 2025 dataset. From the experimental results, it is clear that the CNN-BiLSTM DFS framework improves recall and F1-score compared to other feature selection approaches like MI. This is especially true in imbalanced settings. The decoupling of feature selection from offline and edge-side inference provides a balance between detection accuracy, robustness, and deployability in real-world IIoT settings.

## 1 Introduction

For The expansion of the Internet and the interconnected digital frameworks has significantly increased the complexity and impact of cyber threats. The modern digital frameworks are highly vulnerable to various cyber attacks such as distributed denial of service botnets, reconnaissance missions, and malware that may impact the critical and industrial processes (Abiramasundari and Ramaswamy, 2025). The comprehension and identification of these cyber threats are critical, considering the rising trend of the utilization of interconnected systems in critical infrastructures and the integration of the Industrial Internet of Things and edge computing technologies.

This problem is even more compounded in the IIoT environment, where there are a number of sensors, controllers, and devices that are interconnected as a single network (Qiu et al., 2025). The generated traffic data contains a number of features that are correlated and have temporal dependencies, and there is a need to consider the appropriate features for efficient detection. The Canadian Institute for Cybersecurity (CIC) IIoT 2025 dataset, for example, contains 94 features, and this diversity makes it a significant problem for feature selection.

The Intrusion Detection Systems (IDS) designed for the IIoT environment should be able to effectively represent the relationships between features and identify the temporal patterns of the attacks in the most computationally efficient way possible for deployment at the edge or near the edge devices (Orman, 2025). In recent times, artificial intelligence (AI), particularly deep learning (DL), has been seen to hold tremendous potential for Intrusion Detection Systems (IDS), considering the ability of deep learning to reveal complex and intricate relationships and patterns in huge datasets (Okafor, 2024). Convolutional Neural Networks and Long Short-Term Memory are the most common deep learning techniques that are widely used to reveal the relationships and patterns in the features and the temporal patterns of the attacks in the network data. Though the Intrusion Detection Systems based on deep learning are highly effective in detecting the attacks in the network data, they are mostly challenged by the computational and feature-level transparency issues and the deployment difficulties.

To mitigate the challenges, feature selection (FS) techniques are used to improve the efficiency of the IDS (Hassan et al., 2025). Conventional research on feature selection techniques identifies two main techniques, namely filter and wrapper methods (Guyon and Elisseeff, 2003; Kohavi and John, 1997). The filter methods, such as Mutual Information (MI), are computationally efficient, but they only consider individual features, which fail to consider higher-order non-linear interactions (Akintade et al., 2025). Moreover, filter methods do not consider temporal dependencies in the data, which are common in IIoT environments.

In order to reduce the impact of such issues, the proposed methodology suggests applying a DFS framework that leverages a combination of CNN-BiLSTM model along with permutation importance. As per the proposed approach, the use of CNN is done to extract local interactions among features within short periods of time, while the application of BiLSTM is intended to capture bidirectional temporal dependencies in sequences when performing an offline selection procedure, wherein entire windows are present. The application of bidirectional modeling in this case can be seen to be justified since the aim is not causal packet-by-packet prediction but rather to leverage it to improve the feature ranking process. As the trained edge-side classifier utilizes the extracted feature subset for predictions, it relies only on past instances and hence does not require future observations for classification tasks. It should be noted here that importance of features is evaluated using time-aware perturbation, which essentially captures the influence of disrupting the temporal dependency between features and the output variable. Thus, in essence, this approach is intended to provide feature-level explainability rather than full feature-level transparency.

The key contributions of this study are as follows:

1. A feature selection method that combines a CNN with BiLSTM for selecting deep features from IIoT network traffic data is proposed, taking into account the relationships between the selected features and the temporal nature of IIoT network traffic.
2. A controlled experiment setup is created by employing a constant number of features in the feature set (*K* = 20) and different machine learning classifiers, namely decision trees, k-nearest neighbors (k-NN), logistic regression, naïve Bayes, and random forest, to compare the performance of various feature selection methods. *K* = 20 is applied as an experimental control variable for fair comparisons among various feature selection methods and not necessarily as a claim for global optimality.
3. It is shown that the proposed feature selection method performs better than the existing deep and shallow feature selection methods, which include MI, CNN, and BiLSTM, on the CIC IIoT 2025 dataset with respect to recall and F1 score.
4. A practical approach to classifying IIoT network traffic in which feature selection can be done independently from online traffic classification is proposed.
5. Permutation-based relevance calculation of the features is utilized to allow feature-level transparency; however, full model explainability is beyond the scope of this research.

The rest of this paper is organized in the following manner. In Section 2, we discuss previous work in intrusion detection and feature selection in IoT and IIoT systems. The methodology that we have proposed is discussed in detail in Section 3. The experimental design and results have been discussed in Section 4. An analysis of the results is provided in Section 5, whereas Section 6 concludes the paper with some suggestions for future work.

## 2 State of the art

Intrusion Detection Systems (IDS) have experienced tremendous growth in IoT and IIoT environments as a result of increased intricacy and occurrence of cyber-attacks. The latest advances in deep learning (DL) have allowed for the development of sophisticated detection systems that are capable of detecting sophisticated patterns in network traffic (Alfahaid et al., 2025). This is particularly true for models that include Convolutional Neural Networks (CNNs) and Long Short-Term Memory (LSTM) networks.

Afraji et al. (2025) proposed a deep learning-based approach that incorporates CNN, LSTM, and Gated Recurrent Unit (GRU) layers in order to improve the detection granularity by considering the spatial and temporal features of the network traffic. Likewise, Prakash and Shukla (2025) proposed a privacy-preserving federated learning-based approach that incorporates CNN and BiLSTM in order to improve the detection accuracy while maintaining the privacy of the data in the IoT environment. This demonstrates the significance of privacy-based intrusion detection systems in the context of modern IIoT.

An exhaustive evaluation of different deep learning models in the context of intrusion detection has been performed by Khairullah and Alsenani (2025), which demonstrates the effectiveness of hybrid models that combine CNNs, RNNs, and LSTMs in dealing with the intricacies of IoT security challenges. However, these models mainly emphasize the accuracy of the models and do not consider other factors in the context of their applicability in resource-constrained environments.

Feature selection (FS), however, remains an important tool in improving the efficiency and scalability of IDS systems (Afolabi and Akinola, 2024). In this regard, Sayegh et al. (2024) proposed the integration of LSTM models and the Synthetic Minority Over-sampling Technique (SMOTE) in improving the detection accuracy. In another study, Rihan et al. (2023) proposed hybrid feature selection methods in improving the accuracy of deep learning models in IoT applications. Although these methods have been proven to be effective in improving the accuracy of IDS systems, they do not take into consideration the temporal dependencies in the feature selection process.

Hybrid deep architectures for better learning of the representations have also been a focus of recent studies. In this regard, Zhang et al. (2025) used a CNN-BiLSTM-based model to effectively learn the interactions of features and sequence patterns. The results of this study proved that the use of a combination of architectures can increase the accuracy of the detection. Sharma et al. (2025) proposed a metaheuristic-based approach for the selection of features, utilizing a contraction control factor-based gorilla troop optimizer. This again shows the need for optimization of the features for the purpose of intrusion detection. However, optimization-based approaches are computationally expensive.

Table 1 provides an overview of some of the most significant features of recent intrusion detection techniques in terms of datasets, deep learning architectures, feature selection mechanisms, temporal modeling, feature-level transparency, and edge deployment. From the comparison table, it is clear that most of the existing techniques have either used deep learning or feature selection mechanisms, but not both.

**Table 1:** Comparison between State-of-the-Art intrusion detection approaches and the proposed method.

Study	Dataset	DL architecture	Feature selection (FS)	Temporal modeling	feature-level transparency	Edge deployment	Limitations	Contribution
Afraji et al. (2025)	TON_IoT, CICIDS2017	CNN–LSTM–GRU	None	Yes	No	No	Lack of centralized evaluation and limited deployment analysis	Parallel CNN–LSTM–GRU architecture capturing spatial and temporal features
Prakash and Shukla (2025)	IoT/Fog datasets	CNN–BiLSTM	None	Yes	Limited	Partial (Fog computing)	Limited analysis of feature selection and class imbalance	Privacy-aware federated IDS using hybrid deep learning
Khairullah and Alsenani (2025)	Multi-IoT datasets	DNN/RNN/CNN/LSTM	SMOTE (data-level)	Yes	No	No	Limited real-world validation and lack of feature-level transparency	Comparative analysis of deep learning models for IoT IDS
Sayegh et al. (2024)	CICIDS, NSL-KDD	LSTM	RF + SMOTE	Yes	Limited	No	No support for edge deployment or distributed learning	LSTM combined with RF and SMOTE for improved detection under imbalance
Rihan et al. (2023)	IoT traffic	Hybrid DL	Hybrid FS	Partial	Partial	Partial	Limited support for deployment and scalability	Ensemble feature selection integrated with deep learning
Zhang et al. (2025)	CIC-IDS, BoT-IoT	CNN–BiLSTM–Transformer	Learned FS	Yes	No	No	High computational cost and centralized training	Multi-level modeling of local, temporal, and global patterns
Sharma et al. (2025)	IoT datasets	CCF-GTO + LSTM	Metaheuristic FS	No	Partial	No	Centralized processing and high computational overhead	Bio-inspired feature selection optimization
Proposed Method	CIC IIoT 2025	CNN–BiLSTM	Permutation-based DFS	Yes	Feature-level transparency only	Yes (edge-oriented design)	Binary classification; no external validation; no window-size ablation; no feature-stability analysis	Spatiotemporal feature selection with deployment-oriented lightweight classifiers

Some key observations can be made from this comparison. Firstly, most of the existing works focus on the accuracy of detection, whereas feature-level transparency is neglected. Secondly, only a few works consider deployment issues such as edge or fog computing, which are essential for IIoT scenarios. Finally, most of the existing feature selection methods either do not consider temporal dependencies or use black-box models such as deep models, which do not provide any feature-level transparency. This comparison of the existing works highlights the gap in the existing literature, which is the lack of a unified framework that can consider all the factors such as interactions, temporal dependencies, and feature-level transparency, while also considering deployment issues. The proposed framework addresses this gap by using CNN-BiLSTM-based deep feature selection with permutation importance and lightweight classifiers.

**Figure 1:**
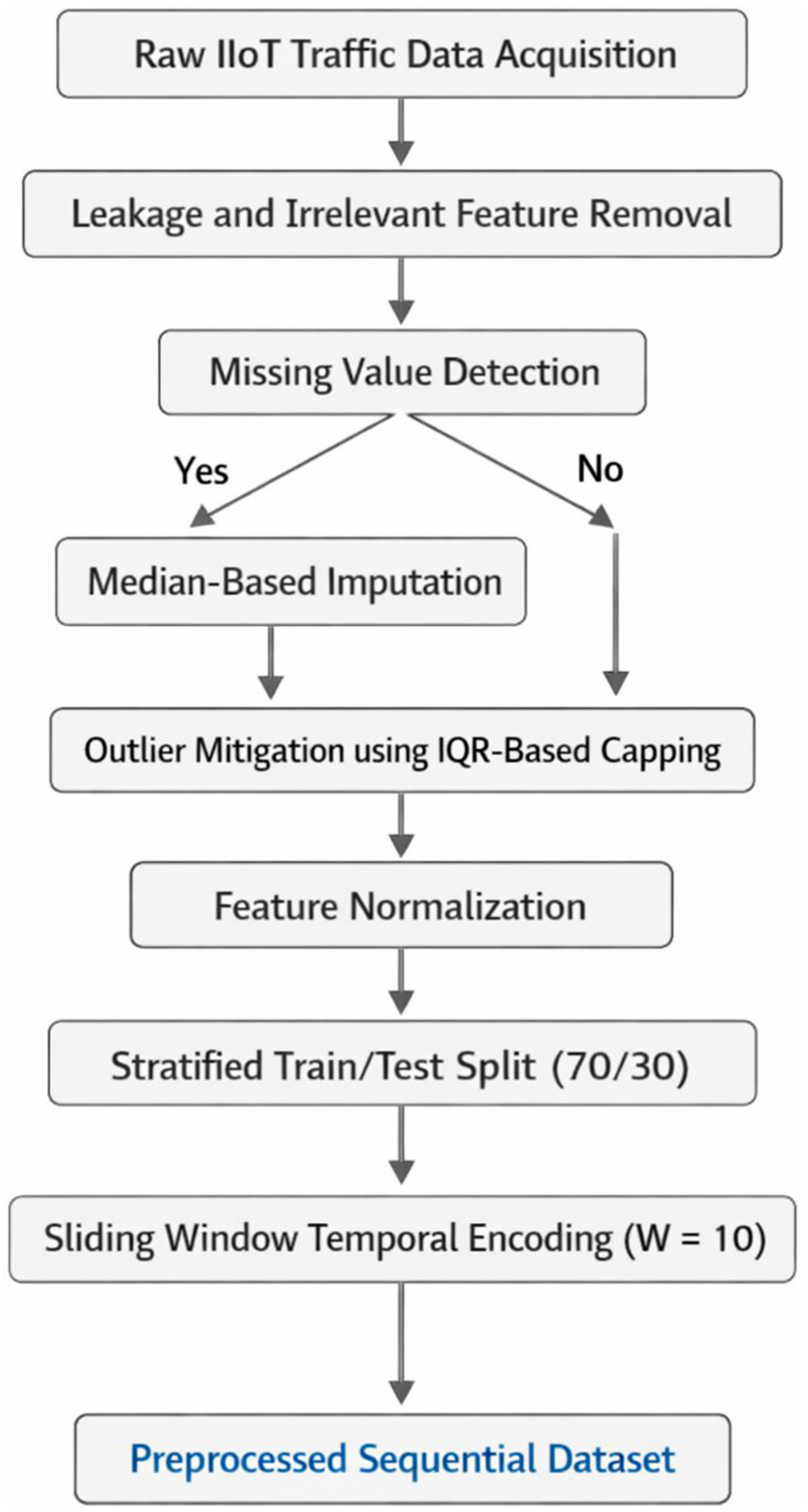
Data preprocessing pipeline.

The proposed data preprocessing and temporal encoding scheme is then applied to the CIC IIoT 2025 dataset. The CIC IIoT 2025 dataset consists of network traffic data, where the observations are of high dimension and are generated in a setting that simulates realistic attack scenarios. The structured data preprocessing pipeline, as shown in Figure 1, is applied before the model. In this pipeline, leakage and uninformative columns are removed to avoid bias in the model. Missing values are then handled through median imputation for robustness. Outliers are optionally handled via an IQR-based capping method. After this, normalization of all numerical features is performed. Subsequently, all numerical features are normalized to stabilize value ranges and improve model convergence.

The dataset is divided into a training set and a testing set, where the size of the training set is 70% and that of the testing set is 30%. To maintain the class distribution of the dataset, a stratified sampling approach is applied. To incorporate time dependence, the tabular data are transformed into overlapping sequences using a sliding window technique, where the size of the window, i.e., *W*, is fixed at 10.

The choice of *W* = 10 reflects a balance between capturing sufficient temporal dependencies and maintaining computational efficiency. Shorter windows may fail to capture sequential attack behavior, whereas larger windows increase computational complexity and may introduce redundant temporal information. While this value has been empirically effective in this study and is supported by prior literature (Wei et al., 2023; Komisarek et al., 2024; Wang and Parish, 2010), no formal sensitivity analysis was conducted, and thus the optimality of this window size is not claimed.

From the literature review, there are indications that deep learning models are efficient for intrusion detection. There are still significant challenges concerning the use of transparent feature selection, temporal ranking, and design-based on deployment. The current framework aims at addressing the problems collectively. But the testing of this framework has been restricted to the use of the CIC IIoT 2025 datasets, and thus, one cannot generalize its applicability to IIoTs.

## 3 Methodology

**Figure 2:**
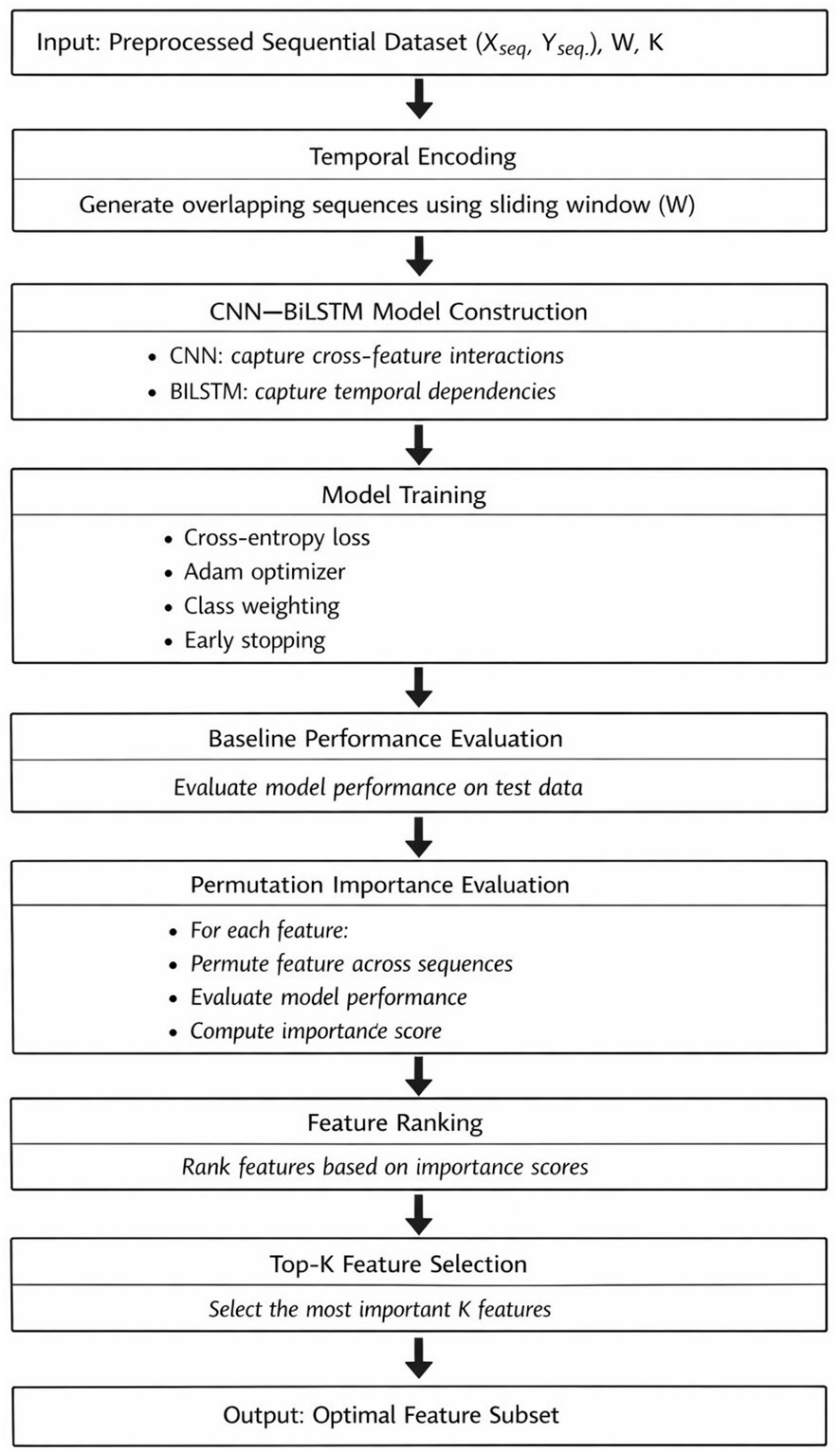
CNN–LSTM DFS Algorithms.

**Figure 3:**
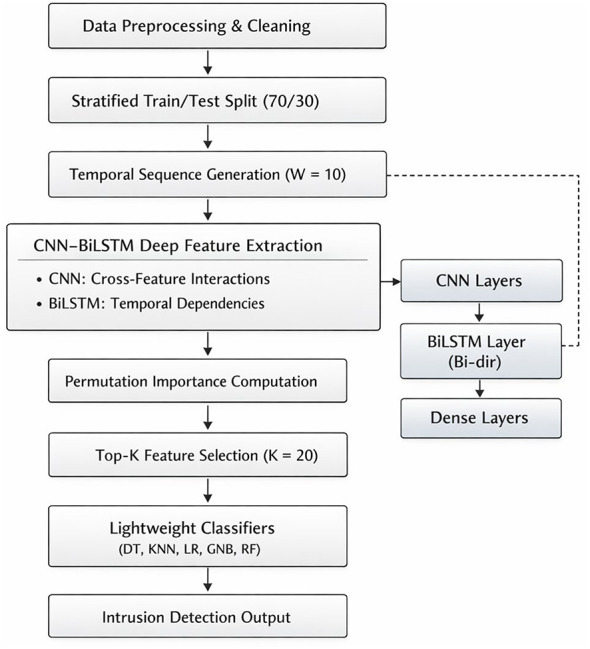
Architecture of the proposed CNN-BiLST FS framework.

The proposed methodology has three major steps: data preprocessing, deep feature selection via the hybrid CNN-BiLSTM structure, and lightweight classification. In order to address the issues of completeness, clarity, and technical rigor in the proposed methodology, all steps of the methodology are clearly stated and mathematically expressed and consistent with the experimental setup. Figure 1 presents the data preprocessing and temporal encoding process, Figure 2 presents the workflow of the DFS process, and Figure 3 presents the structure of the CNN-BiLSTM structure.

### 3.1 Phase 1: data preprocessing and temporal encoding

The CIC IIoT 2025 dataset is preprocessed in order to ensure data quality. To do that, initially, leakage features are intentionally removed in order to avoid any information leakage. Irrelevant features are also removed. Missing numerical data is handled using median imputation. Outliers in the data can be handled using an interquartile range (IQR) capping method. This method limits outliers while maintaining the distribution of the data.

All numerical attributes are normalized to support stable learning and reduce the sensitivity of the learning process to attribute scales. The dataset is subsequently divided into the training and test datasets at a ratio of 70 and 30%, respectively, through stratification, maintaining a balance of 58.44% benign and 41.56% attack instances in both datasets.

For the purpose of handling the time dependency in the network, the network data is converted to sequences by applying the sliding window approach with a window size of *W* = 10. The reason for choosing this number is that it is a middle point between keeping information in the short term and being computationally efficient for the process of offline feature extraction. It is worth noting that no sensitivity analysis or ablation study was carried out on other window sizes. The formal representation of the data transformation from the original table *X*∈ℝ^*N*×*d*^ into sequence data is as follows:

*X*_*seq*_ = {*X*_*t*:*t*+*W*−1_∣*t* = 1, 2, …, *N*−*W*+1}

where each sequence $X_{t:t+W-1}\in {\mathbb{R}}^{W\times d}$ represents a temporally ordered window of observations. This transformation enables the model to recognize sequential patterns in attacks that cannot be represented by static feature representations.

### 3.2 Phase 2: CNN–BiLSTM-based deep feature selection

The second phase involves deep feature selection using a hybrid model of CNN-BiLSTM, as shown in Figures 2, 3. The CNN component of the model is composed of multiple layers of convolutional layers with small kernel sizes to detect local interactions between features within a window, including cross-feature dependencies, which are usually neglected in conventional filter-based methods such as mutual information.

However, since network traffic data in table form is not spatial in the sense of image processing, one-dimensional convolutions still work in identifying local dependencies of neighbor features within a time window. Here “spatial” means the dependency between features at a certain time window, instead of geometrically local. For instance, when there is a flooding attack, there will be changes in the number of packets, packet size and inter-arrival time together. The mathematical representation for the convolution is as follows:


$$\begin{array}{l} H^{\left(l\right)}=\sigma \left(W^{\left(l\right)}*H^{\left(l-1\right)}+b^{\left(l\right)}\right) \end{array}$$

where *H*^(*l*)^ denotes the feature maps at layer *l*, * represents convolution, and σ is a nonlinear activation function. In this context, “spatial” refers to feature-wise relationships at a given time step.

The produced feature maps are then fed into a Bidirectional Long Short-Term Memory (BiLSTM) layer, which processes the sequence in both forward and backward directions:


$$\begin{array}{l} \vec{h_{t}}=LSTM\left(x_{t},\vec{h_{t-1}}\right),\overset{⃖}{h_{t}}=LSTM\left(x_{t},\overset{⃖}{h_{t+1}}\right) \end{array}$$

$$\begin{array}{l} \;h_{t}=\left[\vec{h_{t}};\overset{⃖}{h_{t}}\right] \end{array}$$

Bidirectional recurrence is applied exclusively during the offline deep feature selection phase, where complete temporal windows are available. This enables the model to assess feature relevance by leveraging both past and future contextual information within each sequence. Importantly, this design does not violate causality in practical deployment, since the online inference stage relies solely on lightweight classifiers operating on the selected features and does not require access to future observations.

This enables the model to capture both short-term and long-term temporal dependencies, which is crucial in identifying coordinated and context-dependent attack behaviors. The CNN-BiLSTM model learns an effective spatiotemporal feature representation for IIoT traffic. The model is completed with dense layers and trained with a cross-entropy loss function:


$$\begin{array}{l} L=-\frac{1}{N}\overset{N}{\underset{i=1}{\sum }}\left[y_{i}\log\left({ŷ}_{i}\right)+\left(1-y_{i}\right)\log\left(1-{ŷ}_{i}\right)\right] \end{array}$$

Adam optimization is applied to optimize the network. Early stopping is employed to avoid overfitting and generalize robustly. CNN–BiLSTM model is designed using a one-dimensional convolutional feature extraction network and subsequently feeding the features to a sequential modeling network. First, the CNN part of the model is constructed using a 1D Convolutional layer of 64 filters and a filter size of 3 followed by the application of the ReLU activation function. This is followed by the max-pooling layer that utilizes a pool size of 2 to downsample the input data. The BiLSTM layer has 64 hidden units for both directions of the sequence. In addition, a Dropout layer is employed for the purpose of reducing overfitting. The dropout rate is set to 0.3.

Explicit architectural hyperparameters, such as kernel size, number of filters, number of BiLSTM units, dropout rate, activation function, batch size, learning rate, and epochs, are included in the architecture specification since they affect reproducibility and the capacity of the model. CNN-BiLSTM model was created using one-dimensional time feature extractor and sequential modeler.

### 3.3 Phase 3: permutation importance-based feature ranking

The feature importance is approximated using the permutation-based feature importance test at the raw feature level. For each feature, the feature values are randomly shuffled between the sample sequences while preserving the temporal dependencies within each sequence. This is achieved by permuting the entire time series of the feature of one sequence with the entire time series of the feature of another sequence.

Let M denote the trained CNN–BiLSTM model. The baseline performance is defined as:


$$\begin{array}{l} S_{base}=M\left(X_{seq},Y_{seq}\right) \end{array}$$

After permuting feature *j* the model is re-evaluated:


$$\begin{array}{l} S_{perm}^{\left(j\right)}=M\left(X_{seq}^{\left(j,perm\right)},Y_{seq}\right)\; \end{array}$$

The importance of feature *j* is computed as:


$$\begin{array}{l} I_{j}=S_{base}-S_{perm}^{\left(j\right)} \end{array}$$

This specification provides a measurable and repeatable measure of each variable's importance. Nevertheless, it is worth noting that permutation importance is simply an indicator of the sensitivity of the algorithm to changes in the variables and should thus not be seen as a means for interpreting the whole model but as an indication of *post hoc* feature relevance. To counteract exaggeration, the specification is said to offer feature-level transparency. As an alternative way of explaining the models in future, methods like SHapley Additive exPlanations (SHAP) can also be applied.

Features are ranked according to their importance scores, and the top *K* = 20 features are selected:


$$\begin{array}{l} F_{opt}=TopK\left(I,K\right) \end{array}$$

The selection of *K* = 20 features is used as a controlled experimental setting to ensure fair comparison across all feature selection methods. Given that the original dataset contains 94 features, this represents a reduction of approximately 79%, significantly lowering dimensionality while preserving predictive performance. However, no nested validation or feature stability analysis was performed to determine the optimal value of *K*, and therefore this choice should be interpreted as a standardized evaluation constraint rather than a globally optimal configuration.

### 3.4 Phase 4: lightweight classification and deployment strategy

In the final stage, the chosen set of features is applied to the training process of simple machine learning models. These are logistic regression (LR), decision tree (DT), K-nearest neighbor (KNN), Gaussian naïve Bayes (GNB), and random forest (RF). The purpose of employing logistic regression is to perform linear discrimination between two classes. DT is selected due to its simplicity. KNN represents non-parametric learning using neighborhoods. GNB provides computational performance.

The classifiers are then trained on the reduced features and evaluated for performance metrics, such as accuracy, precision, recall, and F1-score. The class weights are also explicitly used for training the classifiers, addressing the reviewer's concerns of how the imbalances in attack types are handled for efficient detection of minority classes. Decoupling offline deep feature selection from online inference, as proposed in this paper, helps to deploy the proposed framework efficiently, especially in resource-constrained IIoT environments. This decoupling of offline and online aspects of the proposed solution enhances the practical contribution of this study.

## 4 Experiments

This section describes the experimental setup, dataset used, parameters, and evaluation metrics used to evaluate the performance of the proposed framework.

### 4.1 Dataset

The research uses the CIC IIoT 2025 dataset, which is a real-time sensor-based benchmark designed specifically to perform intrusion detection in IIoT environments. This dataset has 685,671 instances and features 94 attributes, which are diverse network traffic characteristics.

The dataset contains approximately 400,711 samples of benign data (58.44%) and 284,960 samples of attack data (41.56%), making it a well-balanced binary classification problem. Nonetheless, in the context of attack types, the dataset is highly unbalanced, with 50 types of attacks. For instance, reconnaissance attacks make up 15.44%, DoS and DDoS attacks make up 8.42 and 8.27%, respectively, whereas brute-force attacks make up only 0.88%.

Scenarios include attacks such as reconnaissance, denial of service (DoS), distributed denial of service (DDoS), man-in-the-middle, web exploitation, brute-force intrusions, and malware. The range of scenarios ensures that the data set represents real-world IIoT threat scenarios while also creating challenges of class imbalance and feature redundancy.

While there are 94 features in the data set, which is not extremely high-dimensional, there is significant redundancy and multicollinearity in the features, making feature selection a non-trivial problem that requires capturing complex feature interactions.

### 4.2 Experimental setup and parameter settings

Before training the model, the data is preprocessed according to the pipeline explained in the Methodology section. This includes the removal of leakage and irrelevant features, imputation of missing values via median statistic-based methods, possible outlier handling via an interquartile range-based approach, and normalization of all numerical features.

The data set is divided into training and testing sets in the proportion of 70 and 30%, respectively, using stratified sampling. To account for the time dependencies in the data, the data is converted into sequences of fixed length *W* = 10 using a sliding-window technique. This value is adopted as a computationally efficient empirical setting for capturing short temporal context; however, no dedicated ablation over alternative window lengths was performed.

The DFS model is constructed based on a hybrid CNN-BiLSTM neural network structure. In the CNN network part, one-dimensional convolution layer with 64 filters, kernel size 3, followed by ReLU activation and max pooling layer with pool size 2 is adopted for dimension reduction. BiLSTM layer includes 64 hidden units in each side for capturing temporal dependencies from two directions. Dropout rate of 0.3 is used after the BiLSTM layer to avoid overfitting problem. The model is trained over 20 epochs with batch size of 32 through Adam optimizer with learning rate of 0.001 and binary cross entropy loss function. The early stopping strategy is used for avoiding overfitting.

For the imbalances between the types of attacks, class weights are integrated into the training process, enabling the model to weigh the classes of attacks appropriately, hence ensuring the effective contribution of the classes to the learning process.

To perform feature selection, the ranking of features is carried out using permutation importance, and the top *K* = 20 features are selected. The use of *K* = 20 serves as a controlled common budget for comparative evaluation across methods, and no nested validation or feature stability analysis was performed to identify an optimal *K* value.

Subsequently, the selected feature subsets are utilized for training a set of light-weight classifiers, namely decision tree (DT), K-nearest neighbor (KNN), logistic regression (LR), Gaussian naïve Bayes (GNB), and random forest (RF), considering a range of modeling assumptions and applicability in resource-constrained IIoT settings.

The implementation process is carried out through several runs of experiments to ensure the stability of the results. The source code used in the study is available upon reasonable request to the corresponding author.

### 4.3 Evaluation measures

For evaluating the performance of the proposed intrusion detection system, classification performance metrics are used, and these metrics are accuracy, precision, recall, and F1-score. Accuracy measures the total number of instances that are correctly classified. Precision measures the number of instances that are correctly classified as attacks out of all instances that are predicted as attacks. Recall measures the number of instances that are correctly predicted as attacks out of all instances that are actually attacks. The F1-score, being the harmonic mean of precision and recall, balances the performance metrics in imbalanced scenarios, as proposed in Owusu-Adjei et al. (2023). All the above metrics will help to assess the proposed intrusion detection technique in great detail, especially under the conditions of imbalance in IIoT environments. Additionally, false-positive rate must be included in case any assertion about false alarms is made, since neither accuracy nor precision, nor recall nor F1 measure this phenomenon.

## 5 Results and discussion

Before presenting the results, it is important to mention that though the CIC IIoT 2025 dataset contains 94 features, it is not an extremely high-dimensional dataset compared to other scenarios in which there are thousands of features. This moderate dimensionality of the dataset allows for a comprehensive evaluation of various feature selection techniques (MI, CNN-Only, BiLSTM-Only, and the proposed hybrid approach) using various classifiers. Therefore, the performance improvements are due to the proposed hybrid approach's capability to capture both cross-feature interactions and temporal relationships, rather than the reduction in dimensionality itself. Filter-based feature selection techniques, such as mutual information (MI), use statistical measures to evaluate individual features.

In this research, MI-based feature selection is used with a non-parametric estimator applicable for classification problems, in which each feature is assigned a score based on its individual importance to the target label. However, this method does not consider interactions between pairs of features or more than that, nor does it consider temporal dependencies in sequential data.

To make the comparison more comprehensive, more experiments are performed based on other deep learning-based feature selection methods. To be more precise, CNN-only feature selection is performed to extract local feature interaction information, and BiLSTM-only feature selection is performed to extract feature information in the time dimension.

The proposed hybrid framework for CNN and BiLSTM is expected to incorporate both modeling of feature interaction and learning of temporal dependencies. The feature importance is also expected to be evaluated through permutation feature importance, which is applicable to the trained model.

**Figure 4:**
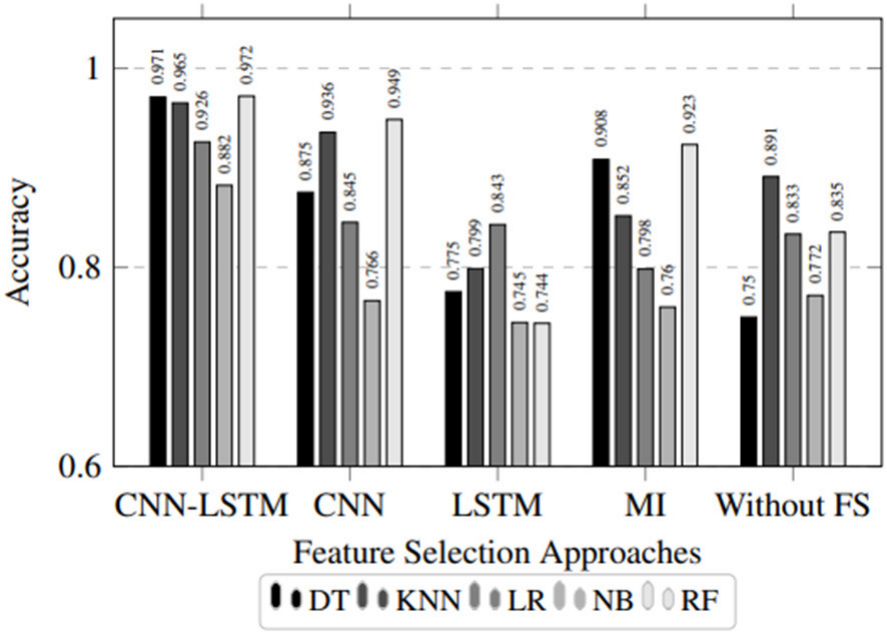
Performance comparison of IDS prediction.

Figure 4 depicts a visual representation of the classification performance using various feature selection techniques, whereas Table 2 represents a detailed quantitative analysis of the performance. It can be observed from the results that the proposed CNN-BiLSTM-based feature selection technique achieves higher recall and F1-score for all classifiers compared to MI, CNN, and BiLSTM alone. The stability of the proposed approach is ensured by the consistent performance in multiple experiments.

**Table 2:** Performance comparison of classifiers with and without feature selection.

Classifier	Metric	CNN–BiLSTM (proposed)	CNN	BiLSTM	MI	Without FS
RF	Accuracy	0.9720	0.9485	0.7438	0.9233	0.8354
	Recall	0.9720	0.8854	0.7219	0.7733	0.4752
	Precision	0.9730	0.9459	0.7427	0.9025	0.5014
	F1-Score	0.9719	0.9133	0.7265	0.8160	0.4842
KNN	Accuracy	0.9651	0.9356	0.7986	0.8517	0.8911
	Recall	0.9651	0.8548	0.8208	0.5900	0.7348
	Precision	0.9663	0.9149	0.8194	0.7309	0.8346
	F1-Score	0.9649	0.8821	0.7985	0.6409	0.7712
DT	Accuracy	0.9710	0.8753	0.7754	0.9083	0.7499
	Recall	0.9710	0.6616	0.7997	0.7698	0.3240
	Precision	0.9718	0.7455	0.8016	0.8328	0.2632
	F1-Score	0.9708	0.6982	0.7754	0.7939	0.2750
LR	Accuracy	0.9259	0.8452	0.8429	0.7983	0.8334
	Recall	0.9259	0.6195	0.8405	0.5346	0.4760
	Precision	0.9297	0.7923	0.8377	0.7604	0.6010
	F1-Score	0.9249	0.6769	0.8390	0.5979	0.5044
NB	Accuracy	0.8824	0.7664	0.7445	0.7600	0.7717
	Recall	0.8824	0.4975	0.7309	0.4438	0.5669
	Precision	0.8836	0.5335	0.7380	0.4412	0.5759
	F1-Score	0.8813	0.4837	0.7334	0.3997	0.5420

When assessing feature selection techniques, it is also essential to differentiate between the impact of feature quality and feature selection. To achieve this, all techniques select an equivalent number of features (*K* = 20). Therefore, any differences in performance will be attributed to feature quality rather than feature quantity. The findings have revealed that the CNN–BiLSTM model is capable of selecting feature subsets that adequately describe spatiotemporal dependencies, which is not adequately achieved by other techniques.

Another factor to consider is that feature importance is a model-dependent attribute. The CNN–BiLSTM model highlights features that are most important for the deep learning model, but these features may not necessarily be optimal for the individual lightweight classifiers. However, the features that are considered are highly transferable for the various classifiers, including DT, KNN, LR, NB, and RF. This implies that the features are fundamental for the data and are useful for a wide range of learning paradigms, making them useful for deployment.

Usually, class imbalance in intrusion detection systems occurs due to the imbalance of the number of samples in various types of attacks rather than between the two classes of benign and malicious. For the CIC IIoT 2025 dataset, though the two classes are well balanced, the number of samples in 50 types of attacks is highly unbalanced. Class weights are used to balance the classes during the training process. The high value of recall for all classifiers, which is above 0.96, suggests that the chosen features are suitable for detecting all types of attacks, whether common or rare.

The experimental results show that the proposed feature selection method can improve detection performance for various classifiers, such as tree-based classifiers, distance-based classifiers, probabilistic classifiers, and linear classifiers. In addition, it is found that the random forest (RF) classifier with CNN-BiLSTM feature selection possesses the highest detection performance in terms of accuracy of 0.9720, recall of 0.9720, precision of 0.9730, and F1-score of 0.9719. Although the false-positive rate (FPR) was not explicitly computed in this study, the high precision (0.9730) suggests a potentially low false alarm tendency. However, without explicit FPR calculation, definitive conclusions regarding false alarm rates cannot be established.

Likewise, the accuracy of the decision tree (DT) classifier is 0.9710, with an F1-score of 0.9708. This shows that even basic models with clear interpretations have high accuracy when trained on feature subsets with significant information. The KNN, LR, and NB classifiers also have high accuracy with features selected by CNN–BiLSTM, which further validates the universality of the suggested approach.

On the contrary, the MI-based feature selection results in low recall and F1 scores, especially for classifiers that utilize feature interaction and temporal patterns. This again shows the weakness of feature evaluation in complex IIoT traffic scenarios, where attacks are likely to be sequential in nature.

To further assess the contribution of individual components, an ablation study is carried out, and the performance of CNN-only, BiLSTM-only, and a combination of CNN and BiLSTM for feature selection is compared. The CNN-only model achieves a high F1-score of 0.9133 on the RF model, indicating that local features are informative but insufficient for the task. The BiLSTM-only model achieves a lower F1-score of 0.7265, indicating that temporal features are insufficient for the task. The combination of CNN and BiLSTM achieves the highest performance, i.e., a high F1-score of 0.9719.

Overall, the proposed CNN-BiLSTM approach for selecting features performs better than the compared approaches because it takes into account not only the intra-window feature relationships, but also the inter-window temporal relationships. The permutation importance technique provides feature-level transparency by identifying which features contribute most to model performance under perturbation. However, it does not provide full model interpretability, as it does not explicitly model internal feature interactions. Fixing the number of selected features at *K* = 20 allows for a fair comparison, yet the robustness analysis was not performed. It is also important to note that even though the proposed model proves effective for the CIC IIoT 2025 data set, the current research results are confined to this particular data set. In other words, these results can be seen as indicative only within the context of the particular data set tested.

### 5.1 Limitations

There are some limitations to this study. Firstly, though the dataset used in the paper contains 94 features, the scalability of the proposed framework for larger IIoT environments with hundreds or thousands of features was not studied. Secondly, the proposed framework was used for binary classification, whereas using it for multi-class intrusion detection would offer deeper insights into the various types of attacks. Finally, though the framework was proposed for deployment on the edge, the inference latency and resource consumption of the proposed framework on real devices were not studied. In addition, there was no study conducted regarding the stability of the suggested feature selection process, or the stability of the selected features based on different resampling schemes. This can be done in future studies by means of measures such as the Jaccard index and Kuncheva index. In addition, although the permutation importance gives some information about the level of features' importance, it still does not give full explanations about the model, as SHAP would.

## 6 Conclusion

In this research paper, a deep feature selection (DFS) methodology is introduced that can be applied for intrusion detection in IIoT using a hybrid CNN-BiLSTM architecture with permutation-based feature relevancy assessment. In this regard, the introduced DFS methodology will overcome the following deficiencies of current feature selection methodologies. On one hand, filter-based feature selection methods, like MI, assess the importance of features in isolation, while on the other hand, the introduced DFS methodology will determine the importance of each feature based on the effect of their time-aware permutation on network performance.

The proposed framework selects the most appropriate features, which can then be used to train lightweight classifiers, making it an effective tool for intrusion detection in IIoT environments. The experimental results using the CIC IIoT 2025 dataset demonstrate the effectiveness of the proposed DFS framework, which is based on the hybrid CNN-BiLSTM model, in improving the performance of various classifiers in the context of intrusion detection. For instance, the proposed framework improves the recall and F1-score of the classifiers, thus making them more effective in detecting intrusion in the IIoT environment with lower false alarm tendency.

The proposed framework is also deployment-oriented in its structure because of the separation of the feature selection process from the inference phase. This allows achieving better scalability of the solution in edge settings while still maintaining a high level of detection ability. Another advantage is that the usage of a smaller set of features contributes to computational efficiency of the deployment phase despite the absence of specific latency/resource-consumption data in the current research.

The difference between feature importance and model explainability should be emphasized. While the presented solution enables identifying the most important features for prediction with regard to their effect on the accuracy score after permutation-based manipulation, the interaction of such features in the model cannot be fully understood with the current techniques.

While the CIC IIoT 2025 dataset may reflect practical IIoT traffic situations, the assessment is only carried out using one benchmark. As such, although the steady increase in performance through various classifiers and baseline feature selections suggests that the framework is reliable with this dataset, its applicability to other IIoT datasets must be understood with reservations. The wide range of attacks in the data set (50 classes) and the existence of class imbalance in the data set somewhat justify the reliability of the framework, but further verification on other benchmarks is still required.

Potential directions for future research involve generalization of the above technique for use in multiclass intrusion detection, adaptation of feature selection based on concept drift, and the application of this framework to federated learning models. Furthermore, potential directions for future research will include external benchmarking of this algorithm against other IoT and IIoT intrusion detection systems, analysis of sensitivity of this model to window size and size of selected features, evaluation of the stability of the feature selection process with varying resamples, and false positive rate.

## Data Availability

Publicly available datasets were analyzed in this study. The data used in this study are publicly available from the CIC IIoT 2025 dataset repository.
